# Breast metastasis in high-grade endometrial stromal sarcoma (HG-ESS) with BCOR rearrangement: a case report of a novel metastatic site

**DOI:** 10.1186/s13000-023-01313-z

**Published:** 2023-03-02

**Authors:** Ashlyn Fong, Saul Offman

**Affiliations:** 1 Department of Pathology, Dalhousie University, Halifax, NS Canada; 2 Division of Anatomic Pathology, Nova Scotia Health, Halifax, NS Canada

**Keywords:** Endometrial stromal sarcoma, Endometrial stromal sarcoma classification, Endometrial neoplasms, Gene fusion, *BCOR* protein

## Abstract

**Background:**

The *ZC3H7B-BCOR* fusion gene has recently been described in tumours with kinship to so-called high grade endometrial stromal sarcoma (HG-ESS). This subset of tumour behaves similarly to *YWHAE-NUTM2A/B* HG-ESS, however, they are both morphologically and immunophenotypically distinct neoplasms. The identified rearrangements in the *BCOR* gene have been accepted as both the driver and requisite feature in creating a novel sub-entity within the category of HG-ESS.

Preliminary investigations into *BCOR* HG-ESS have shown similar outcomes to *YWHAE-NUTM2A/B* HG-ESS, with patients typically presenting with high stage disease. Clinical recurrences and metastases to lymph nodes, sacrum/bone, pelvis/peritoneum, lung, bowel and skin have been identified. In this report, we describe a case of *BCOR* HG-ESS, that is deeply myoinvasive and widely metastatic. Metastatic deposits include a mass in the breast discovered on self-examination; a metastatic site that has yet to be reported in the literature.

**Case presentation:**

A 59-year-old female underwent biopsy for post-menopausal bleeding, yielding a diagnosis of “low-grade spindle cell neoplasm with myxoid stroma and endometrial glands”, favouring endometrial stromal sarcoma (ESS). She was then referred for total hysterectomy and bilateral salpingo-oophorectomy. The resected uterine neoplasm was both intracavitary and deeply myoinvasive with morphology consistent with that of the biopsy specimen. Characteristic immunohistochemistry (IHC) was noted, and fluorescence in situ hybridization confirmed *BCOR* rearrangement, supporting a diagnosis of *BCOR* HG-ESS. A few months postoperatively, the patient underwent needle core biopsy of the breast which revealed metastatic HG-ESS.

**Conclusions:**

This case highlights some of the diagnostic challenges posed by uterine mesenchymal neoplasms, and exemplifies the emerging histomorphologic, immunohistochemical, molecular and clinicopathologic features of the recently described HG-ESS with *ZC3H7B-BCOR* fusion. It adds to the body of evidence supporting the inclusion of *BCOR* HG-ESS as a sub-entity of HG-ESS within the endometrial stromal and related tumours subcategory of uterine mesenchymal tumors, as well as the poor prognosis and high metastatic potential of this tumor.

## Background

The identification of specific genetic rearrangements and aberrations has led to evolutions in the classification of uterine mesenchymal neoplasms. One such fusion gene (*ZC3H7B-BCOR*) has recently been described in tumours with kinship to so-called high grade endometrial stromal sarcoma (HG-ESS) [1–3].

The World Health Organization (WHO) classification of tumours (2020) [4–7] includes four diagnostic entities within the endometrial stromal and related tumours subcategory; endometrial stromal nodule (ESN), low-grade endometrial stromal sarcoma (LG-ESS), HG-ESS and undifferentiated uterine sarcoma (UUS) (in addition to “uterine tumour resembling ovarian sex cord tumour”). HG-ESSs were recently redefined following the detection of the novel *YWHAE-NUTM2A/B* fusion gene associated with clinically aggressive tumours [1]. Subsequently, the *ZC3H7B-BCOR* fusion gene was discovered in a subset of tumours that behave similarly to *YWHAE-NUTM2A/B* HG-ESS, however they are both morphologically and immunophenotypically distinct neoplasms. The identification of this transcript has been accepted as both the driver and requisite feature in creating a novel sub-entity within the category of HG-ESS [1–3]. In addition to the aforementioned molecular abnormalities, the WHO classification of tumours includes *BCOR* internal tandem duplications (ITD) as a defining molecular signature for HGESS [6]. Other rarely described fusions include *EPC1-BCOR, JAZF1-BCORL1* and *BRD8-PHF* [6].

Preliminary investigations into *BCOR* HG-ESS have shown that patients appear to have similar outcomes to those with *YWHAE-NUTM2A/B* HG-ESS, and tend to present with high stage disease [3]. Clinical recurrences and metastases to the lymph nodes, sacrum/bone, pelvis/peritoneum, lung, bowel and skin have all been identified [1–3]. In this report, we describe a case of *BCOR* HG-ESS that presented as a deeply myoinvasive tumour with widespread metastatic disease. The latter includes a deposit in the breast discovered on self-examination; a metastatic site that has yet to be knowingly reported in the literature.

## Case presentation

A 59-year-old female presented with a 6 month history of post-menopausal bleeding. Transvaginal ultrasound revealed markedly thickened endometrium suspicious for malignancy. She underwent hysteroscopy with dilatation and curettage which yielded a diagnosis of “low-grade spindle cell neoplasm with myxoid stroma and endometrial glands”. Endometrial stromal sarcoma (ESS) (with glands) was favoured, although adenosarcoma could not be ruled out. She was then referred to gynecologic oncology for total hysterectomy and bilateral salpingo-oophorectomy. The surgery was complicated by dense bladder adhesions, and multiple suspicious pelvic lymph nodes were identified intraoperatively. Upon completion, there was no obvious disease remaining in the pelvis.

Post-operative computed tomography (CT) revealed bilateral pulmonary nodules suspicious for metastatic deposits. Follow up CT showed widespread metastatic disease affecting both lungs, the vertebral column, bilateral ilia, bilateral femoral diaphyses and retroperitoneal lymph nodes. Additionally, prior to gynecologic surgical intervention, the patient self-palpated a right breast lump. Physical examination at the time revealed no concerning abnormalities, but subsequent mammogram showed heterogeneous features, with diffuse cortical thickening and micro-lobulated margins; risk assessment was determined to be BiRADS category 4C. Breast core biopsies were taken, revealing metastatic disease in the breast.

## Gross appearance

The uterine cavity was filled with multiple tan-grey, soft, fleshy endometrial masses ranging from 2.4–6.5 cm in greatest dimension. The largest mass had a myxoid appearance and was located in the left posterolateral wall. There was gross extension to the resection margin posteriorly.

## Microscopic description and differential diagnosis

An initial endometrial biopsy was composed predominantly of blood with strips of inactive endometrium. Associated cytology showed atypical glandular cells. This finding, along with the markedly thickened endometrium seen on transvaginal ultrasound, prompted recommendation for repeat sampling. Repeat biopsy showed a low-grade spindle cell neoplasm with myxoid stroma and admixed endometrial glands. Cellularity was variable and occasional mitoses were identified. Endometrial glands showed no cytologic atypia. Immunohistochemical (IHC) studies were performed and revealed that the spindle cells stained positively for estrogen receptor (ER) and CD10. Desmin, muscle specific actin (MSA), alpha-smooth muscle actin (SMA) and p16 were all negative. Wild-type p53 expression was observed and alcian blue highlighted abundant stromal mucin. The principal differential diagnosis at this juncture included low-grade Mullerian adenosarcoma, ESN, and LG-ESS. Definitive surgical management was recommended.

**Fig. 1:**
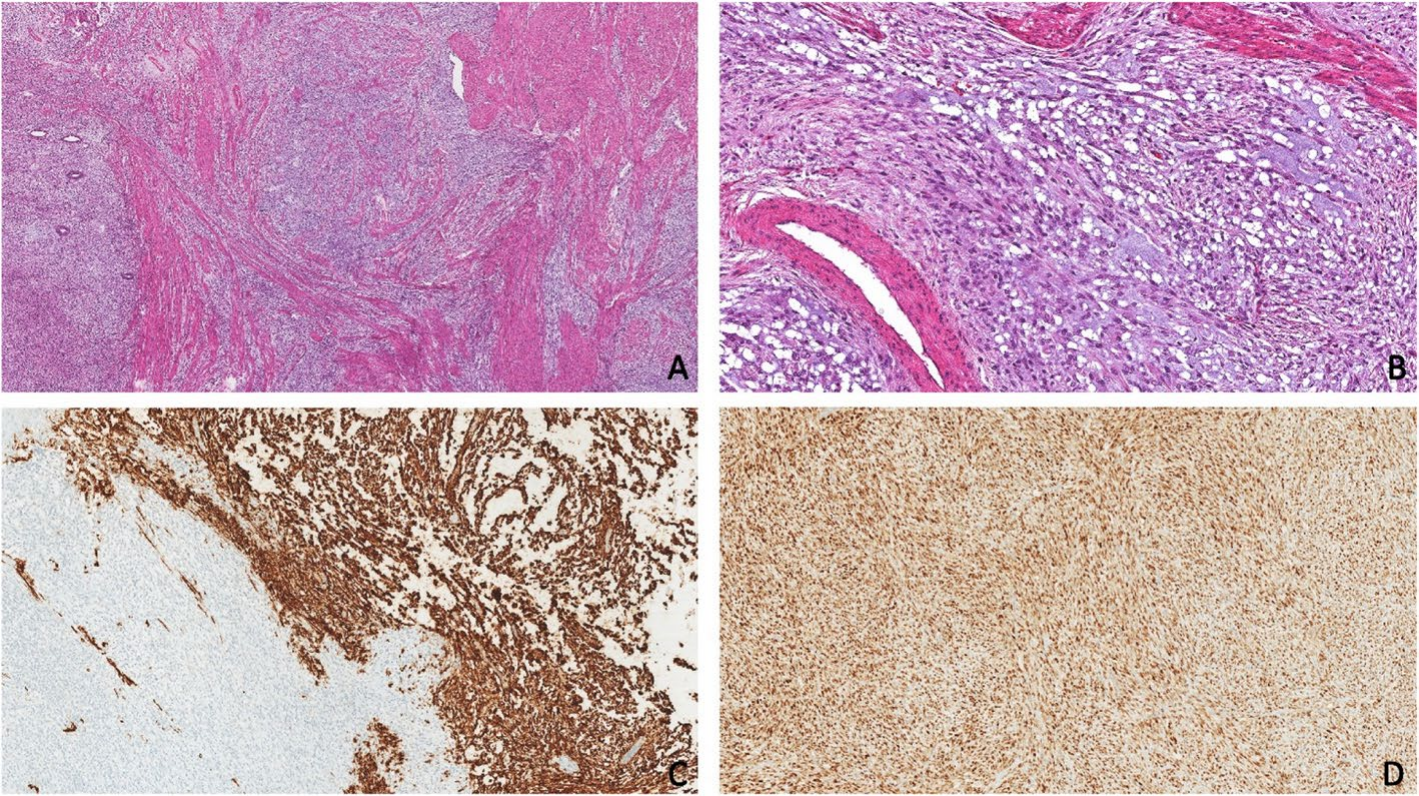
*BCOR* HG-ESS from hysterectomy specimen. **A** H&E section of endomyometrium at 40X magnification showing fascicular growth pattern of *BCOR* HG-ESS set within a myxoid stroma. Incorporated benign endometrial glands are seen at left. **B** H&E section of endomyometrium at 200X magnification showing atypical spindle cells arranged in short fascicles set within a fibromyxoid stroma. **C** CD10 immunohistochemistry at 100X magnification. Note the zones of strong/diffuse expression (right) and adjacent negative population of neoplastic cells (left). **D** Cyclin D1 immunohistochemistry showing strong/diffuse expression at 100X magnification

Hysterectomy and bilateral salpingo-oophorectomy was performed. The neoplasm was both intracavitary and deeply myoinvasive with extension to the resection margin. The morphology again included atypical spindle cells, arranged in short fascicles and set within a myxoid stroma (Fig. 1). Mitotic activity was high (> 20 mitoses per 10 high power fields) and necrosis was identified within the intracavitary component. Ovarian and fallopian tube metastases were noted, as was lymphovascular invasion. Retroperitoneal lymph nodes were also involved. No atypical glands, phyllodes-like architecture or periglandular cuffing to suggest adenosarcoma could be identified, nor any morphology suggesting conventional LG-ESS. IHC was positive for CD10 (both strong/diffuse and negative zones were noted), cyclin D1 (strong/diffuse), cKIT, beta catenin (nuclear), vimentin and MDM2. Estrogen receptor (ER), progesterone receptor (PR), desmin, MSA, SMA, WT1, CD34, DOG1, p16, S100, HMB45, AE1/AE3 and ALK-5A4 were all negative. There was wild-type p53 expression and weak variable MART1 staining.

Although the differential diagnosis was broad and included myxoid leiomyosarcoma (LMS), inflammatory myofibroblastic tumour, gastrointestinal stromal tumour (GIST), melanoma, adenosarcoma, and malignant mixed Mullerian tumour (MMMT), the histomorphologic and IHC features were most in keeping with a fibromyxoid variant of endometrial stromal sarcoma (ESS). The high mitotic activity, advanced stage disease, and strong cyclin D1 expression suggested HG-ESS as the top differential. Because HG-ESS variants can be associated with specific translocations (e.g. *YWHAE-NUTM2A/B* and *ZC3H7B-BCOR*), this case was referred out for fluorescence in situ hybridization (FISH) testing to Ashion ATGen Clinical Laboratory in Phoenix, Arizona. Rearrangement of the *BCOR* (Xp11.4) locus was identified. The positive *BCOR* translocation supported the diagnosis of *BCOR* HG-ESS. Notably, *BCOR* HG-ESS has been proposed to have an association with *MDM2* amplification [4]. Although MDM2 expression was noted with positive IHC in this case, the tumour was negative for amplification of the *MDM2*(12q15) locus by FISH.

**Fig. 2:**
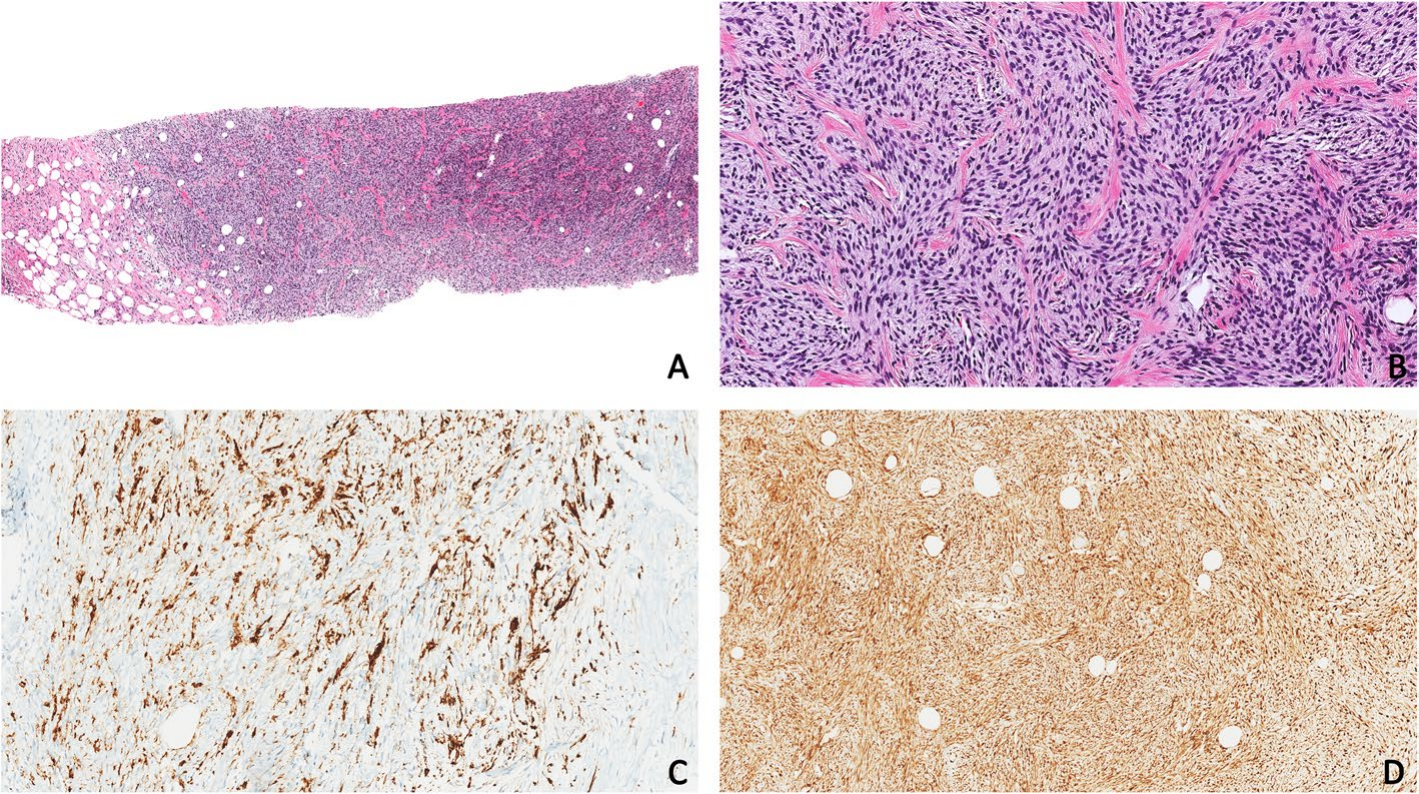
*BCOR* HG-ESS from breast needle core biopsy. **A** H&E section of breast needle core biopsy with metastatic *BCOR* HG-ESS at 40X magnification, showing moderately cellular spindle cell lesion with no normal mammary parenchyma. **B** H&E section of breast needle core biopsy with metastatic BCOR HG-ESS at 200X magnification, showing a mitotically active, spindle-cell lesion arranged in short fascicles within a predominantly fibrous stroma. **C** CD10 immunohistochemistry at 200X magnification. Note cells with strong/diffuse expression admixed with those with negative expression. **D** Cyclin D1 immunohistochemistry showing strong/diffuse expression at 100X magnification

A few months postoperatively, the patient underwent breast core biopsy for a 10 mm mass at the 12 o’clock position of the right breast. Sections showed a moderately cellular, mitotically active, spindle-cell lesion arranged in short fascicles within a fibromyxoid stroma (Fig. 2). Normal mammary glandular parenchyma was not present. IHC showed positive expression of CD10 (both strong/diffuse and negative zones), cyclin D1 (strong/diffuse), CKIT, beta catenin (nuclear), and MDM2. S100 and p63 were weakly and focally positive. Negative staining was observed with desmin, MSA, AE1/AE3, CK5/6, CD34, HMB45 and ERG. Histomorphologic and IHC features were also compared to the hysterectomy specimen. Collectively, the features supported a diagnosis of metastatic HG-ESS.

## Treatment

Initial treatment in this case included docetaxel and gemcitabine chemotherapy. There was progression of disease following 3 cycles, after which therapy was changed to doxorubicin. Disease progression stabilized after initiation of doxorubicin chemotherapy. Palliative radiation therapy was undertaken for spinal and cranial metastases and the patient was transferred to hospice care.

## Discussion

This case highlights some of the diagnostic challenges generally posed by uterine mesenchymal neoplasms, and exemplifies the emerging histomorphologic, IHC, molecular and clinicopathologic features of the recently described HG-ESS with *ZC3H7B-BCOR* fusion gene. Gross and microscopic features of this tumour leave a broad differential and multiple ancillary tests are required to arrive at a diagnosis of *BCOR* HG-ESS.

Endometrial stromal sarcomas (ESS) account for 7–25% of uterine sarcomas and about 1% of all malignant uterine neoplasms [1]. These mesenchymal tumours have undergone numerous revisions to their classification system since their initial designation in 1959, more recently due to the increased understanding of their molecular genetics [1].

The gross and histomorphologic features of ESN and LG-ESS show significant overlap. Both present as intramural or submucosal masses with a soft, tan-yellow cut surface [1, 4, 5]. Gross features that may differentiate them include evidence of myometrial invasion or intravascular tumour plugs, as seen in LG-ESS, but not ESN [4, 5]. Additionally, LG-ESS may present as poorly-defined, coalescent nodules [5]. Necrosis, hemorrhage and/or cystic degeneration can be identified in both [1, 4, 5]. Microscopic examination shows small, round cells with round to oval nuclei and scant cytoplasm, akin to proliferating endometrial stromal cells [1, 4, 5]. ESN can be distinguished microscopically from LG-ESS by its relative circumscription (≤ 3 myometrial projections, each no more than 3 mm from the lesional border), and absence of lymphovascular space invasion [4]. ESNs also preferentially show a distinctive concentric arrangement of cells around thin-walled arterioles [1, 4, 5]. Myometrial invasion (beyond the limits stated for ESN), and lymphovascular space invasion are features diagnostic of LG-ESS [5]. Therefore, in toto sampling of the tumour/myometrial interface is required to ensure lack of invasion to reliably exclude LG-ESS [1, 5]. Mitotic activity in LG-ESS is typically < 5 mitotic figures/10 high power fields (hpf), but higher mitotic activity has been reported [1]. IHC shows positive staining with CD10, ER and PR [1, 5]. Nuclear beta catenin positivity is seen in > 50% of cases, and cKIT (CD117) may be focally positive [1, 5]. DOG1 is typically negative [1]. LG-ESS may also show divergent immunostaining within potential areas of smooth muscle or sex-cord differentiation. One diagnostic challenge for ESN/LG-ESS is distinguishing them from highly-cellular leiomyoma (HCL). Generally, ESN/LG-ESS will have haphazard growth and/or shorter fascicles and thin-walled arterioles, as compared to highly-cellular leiomyoma, which has longer fascicles and thick-walled blood vessels [1, 2]. IHC is less helpful due to the possibility of smooth muscle differentiation in ESN/ESS, although a desmin positive/CD 10 negative result favors HCL [1, 2].

HG-ESS (with *YWHAE* rearrangement) are characterized by small, round cells with cytologic atypia that is marked, but does not reach threshold for UUS [1, 6]. A low-grade, spindled cell component can be identified in the majority of cases [1, 6]. Grossly, tumours are variable in size with reports up to 12 cm [1]. They show extensive myometrial invasion and are classically tan-pink to tan-grey and fleshy on cut surface [1, 6]. The round cells are most commonly found in nests with scant eosinophilic cytoplasm and are surrounded by arborizing blood vessels [1, 6]. The spindled cell component is typically moderately cellular, cytologically low-grade, and may be fibrous, fibroelastotic or fibromyxoid [1, 6]. Lymphovascular and myometrial invasion are typically readily identified. Mitotic activity is high with most tumours displaying > 10 mitoses/10 hpf [1, 6]. These tumours have high metastatic potential, with reports of metastatic deposits consisting of the round cell component, spindled cell component, or both [1, 6]. The round cells do not express CD10, ER or PR, but display strong staining for cyclin D1 and cKIT [1, 6]. Wild-type p53 expression is also noted [1, 6].

The majority of HG-ESS with *BCOR* re-arrangement will also stain positively for cyclin D1 [1–3, 6]. In contrast to other HG-ESS, they typically retain strong CD10 expression [1–3, 6] and variable ER and PR staining [1, 6]. Other distinguishing features of *BCOR* HG-ESS include longer fascicular growth, and the presence of a distinct myxoid matrix less commonly seen in *YWHAE* HG-ESS [1, 3, 6]. Recent literature has also identified *MDM2* amplifications in association with *BCOR* HG-ESS [8]. Although not identified in the case herein, this finding is uncommon in other uterine mesenchymal neoplasms and may provide a possible diagnostic adjunct for *BCOR* HG-ESS [8]. The fascicular growth and myxoid stroma seen in *BCOR* HG-ESS can make it difficult to distinguish from myxoid LMS in particular. That being said, the nuclei in myxoid LMS tend to be cytologically bland and mitotic activity is minimal [2]. These will also typically show IHC expression of desmin, smooth muscle actin or caldesmon [1–3]. Absence of staining with these smooth muscle markers should prompt consideration of *BCOR* HG-ESS in a case being considered for myxoid LMS [1, 2].

The rarity of these tumours leaves limited information to guide prognosis and treatment. Recurrences and metastases within 5 years of diagnosis have been identified in many cases of both *YWHAE* HG-ESS and *BCOR* HG-ESS [1–3]. In the initial case series describing *BCOR* HG-ESS, three tumours were analyzed, each with aggressive clinical disease [2]. Metastases to the bone, lung, skin and peritoneum were all described [2]. A report on 17 additional cases reviewed follow-up data for 5 patients [3]. Each of these patients had recurrence or metastasis within 80 months of the initial diagnosis [3]. Metastatic sites described in the literature thus far have included lymph nodes, sacrum/bone, pelvis/peritoneum, lung, bowel and skin [1–3]. The case described herein identifies an example of *BCOR* HG-ESS with widespread pelvic, bony and lung disease, as well as a novel site of metastasis to the breast. This supports previous evidence suggesting HG-ESS with *BCOR* rearrangement to be clinically aggressive and therefore similar to *YWHAE* HG-ESS.

This case adds to the body of evidence supporting the recent inclusion of *BCOR* HG-ESS as a sub-entity of HG-ESS within the endometrial stromal and related tumours subcategory of uterine mesenchymal tumors, as well as the poor prognostic implications and high metastatic potential of this tumor.

## Data Availability

Not applicable.

## Abbreviations

ESS: Endometrial stromal sarcoma
LG-ESS: Low grade endometrial stromal sarcoma
HG-ESS: High grade endometrial stromal sarcoma
ESN: Endometrial stromal nodule
UUS: Undifferentiated uterine sarcoma
WHO: World Health Organization
CT: Computed tomography
BiRADS: Breast imaging reporting and data system
IHC: Immunohistochemical, immunohistochemistry
ER: Estrogen receptor
LMS: Leiomyosarcoma
GIST: Gastrointestinal stromal tumour
MMMT: Malignant mixed Mullerian tumour
HCL: Highly-cellular leiomyoma
FISH: Fluorescence in situ hybridization
hpf: High power fields
